# Musical abilities in children with developmental cerebellar anomalies

**DOI:** 10.3389/fnsys.2022.886427

**Published:** 2022-08-18

**Authors:** Antoine Guinamard, Sylvain Clément, Sophie Goemaere, Alice Mary, Audrey Riquet, Delphine Dellacherie

**Affiliations:** ^1^Univ. Lille, ULR 4072 – PSITEC – Psychologie: Interactions, Temps, Émotions, Cognition, Lille, France; ^2^CHU Lille, Centre de Référence Malformations et Maladies Congénitales du Cervelet, Lille, France; ^3^CHU Lille, Centre Régional de Diagnostic des Troubles d’Apprentissage, Lille, France

**Keywords:** cerebellum, developmental cerebellar anomalies, ataxia, music perception, music production, singing, children, rhythm

## Abstract

Developmental Cerebellar Anomalies (DCA) are rare diseases (e.g., Joubert syndrome) that affect various motor and non-motor functions during childhood. The present study examined whether music perception and production are affected in children with DCA. Sixteen children with DCA and 37 healthy matched control children were tested with the Montreal Battery for Evaluation of Musical Abilities (MBEMA) to assess musical perception. Musical production was assessed using two singing tasks: a pitch-matching task and a melodic reproduction task. Mixed model analyses showed that children with DCA were impaired on the MBEMA rhythm perception subtest, whereas there was no difference between the two groups on the melodic perception subtest. Children with DCA were also impaired in the melodic reproduction task. In both groups, singing performance was positively correlated with rhythmic and melodic perception scores, and a strong correlation was found between singing ability and oro-bucco-facial praxis in children with DCA. Overall, children with DCA showed impairments in both music perception and production, although heterogeneity in cerebellar patient’s profiles was highlighted by individual analyses. These results confirm the role of the cerebellum in rhythm processing as well as in the vocal sensorimotor loop in a developmental perspective. Rhythmic deficits in cerebellar patients are discussed in light of recent work on predictive timing networks including the cerebellum. Our results open innovative remediation perspectives aiming at improving perceptual and/or production musical abilities while considering the heterogeneity of patients’ clinical profiles to design music-based therapies.

## Introduction

Developmental Cerebellar Anomalies (DCA) are rare diseases characterized by a quantitative and/or qualitative abnormality of the cerebellum that is present at birth or manifests in early childhood, and persists into adulthood. These abnormalities in most cases lead to the presence of congenital cerebellar ataxia, which refers to a lack of coordination and balance disorders occurring from birth or in the first months of life. Initially manifested by the presence of hypotonia and psychomotor retardation, cerebellar ataxia may improve or remain stable during development, without regression. The most well-known congenital ataxia is Joubert syndrome, a genetic disorder characterized by a specific cerebellar malformation known as the “molar tooth sign” on brain imaging. Other congenital ataxias are associated with hypoplasia, dysgenesis, or atrophy of the cerebellar vermis and/or cerebellar hemispheres or brainstem. Brain imaging may, however, appear normal, and in many cases the etiology of these congenital cerebellar ataxias remains unknown (Steinlin et al., 1998; Garel et al., 2011; Musselman et al., 2014; Bertini et al., 2018). In addition to the well-known motor (balance and coordination) disorders caused by cerebellar motor syndrome, children with DCA may have a variety of cognitive and socio-emotional deficits affecting non-motor functions (Schmahmann and Sherman, 1998; Steinlin et al., 1999; Tavano et al., 2007). Among these deficits, timing abilities may be affected in these patients, particularly rhythmic abilities (Dennis et al., 2004, 2009; Hopyan et al., 2009; Bégel et al., 2022a), but it is not known whether musical abilities are more generally affected, and whether these difficulties affect only musical perception or whether production disorders are also found. However, there is growing evidence that the development of musical skills is important for the development of higher functions (Flaugnacco et al., 2014; Lense et al., 2021). A better understanding of the impact of childhood cerebellar pathologies on musical abilities should lead to improved diagnosis and remediation of DCA patients.

Musical perception and production abilities emerge early and develop spontaneously in children (Winkler et al., 2009; Perani et al., 2010; Stadler Elmer, 2012; Provasi et al., 2014a), such that the majority of the general population have sophisticated music perception abilities (Bigand, 2003) and are able to sing in tune and in time, without formal musical training (Dalla Bella et al., 2007; Dalla Bella and Berkowska, 2009; Berkowska and Dalla Bella, 2013). Musical ability is comprised of complex skills and involves a distributed brain network, including the cerebellum (Peretz and Zatorre, 2005; Chen et al., 2008; Herholz and Zatorre, 2012; Herholz et al., 2012).

The involvement of the cerebellum is reported in various aspects of musical perception and production. While for a long time the cerebellum was mainly considered to play a role in motor functions, theories have evolved in recent decades to include its role in higher cognitive functions (Schmahmann and Sherman, 1998; Schmahmann, 2019) and in more fundamental tasks related to sensory functions (Baumann et al., 2015). Thus, the cerebellum is active in purely sensory auditory processing and is involved in both passive listening and pitch discrimination tasks, especially as the difficulty of the task increases (Parsons et al., 2009; Petacchi et al., 2011; Lega et al., 2016). In addition to pitch processing, sound duration processing also engages the cerebellum (Belin et al., 2002), and cerebellar gray matter volumes are positively related to beat interval discrimination abilities (Paquette et al., 2017). It is therefore not surprising that neuroimaging studies have demonstrated its activation during music listening (Brown et al., 2004b). For instance, listening to musical rhythms, even passively, recruits the cerebellum along with other motor regions of the brain (Chen et al., 2008; Gordon et al., 2018) and cerebellar activations are greater during rhythmic than during melodic tasks (Parsons, 2001; Thaut et al., 2014). Some studies suggest also that the cerebellum has a role in working memory for rhythm (Jerde et al., 2011; Konoike et al., 2012; Teki and Griffiths, 2016) whereas its involvement in melodic working memory has not been reported to date.

Like music perception, music production involves several processes that rely in part on the cerebellum. Whether one is singing or playing an instrument, producing the desired pitch sequence and rhythmic structure requires precise motor control and auditory-motor integration, with feedforward and feedback components, which in turn involves the cerebellum (Wolpert et al., 1998; Bastian, 2006; Zatorre et al., 2007; Kleber and Zarate, 2014; Johnson et al., 2019; Peng et al., 2021). Neuroimaging studies have shown that the networks involved in singing and playing an instrument partially overlap (Segado et al., 2018) and in both cases have shown that the cerebellum is activated (Zatorre et al., 2007; Zarate and Zatorre, 2008; Kleber and Zarate, 2014; Brown et al., 2015; González-García et al., 2016). The cerebellum is involved in motor rhythm reproduction tasks, especially when rhythms are complex or novel (Penhune et al., 1998) and it is particularly active when musicians make and correct errors while performing (Pfordresher et al., 2014). Singing single notes (Perry et al., 1999) as well as short melodies with or without words also activates the cerebellum (Brown et al., 2004a; Kleber et al., 2007), and the more experienced the singer, the more active the cerebellum becomes during singing (Kleber et al., 2010). Singing seems to involve especially the lobule VI of the posterior cerebellum, which somatotopically represents the lips and tongue and is also activated during speech (Callan et al., 2007). These activations are not surprising knowing that the cerebellum is involved in motor control of vocal muscles (Grabski et al., 2012). More broadly, the cerebellum is involved in the vocal sensorimotor loop (Berkowska and Dalla Bella, 2009), with a crucial role in motor-to-auditory and self-initiated sounds predictions (Knolle et al., 2012, 2013) and in auditory feedback control (Li et al., 2019; Peng et al., 2021).

One of the cerebellum’s roles common to both musical perception and production is its participation in timing processes. The cerebellum is part of a distributed neural network, including the basal ganglia, the motor cortex and the premotor cortex, all of which enable temporal processing (Coull and Nobre, 2008; Coull et al., 2011; Bareš et al., 2018). The cerebellum has a recognized role in absolute duration-based timing (Grube et al., 2010b; Teki et al., 2011; Breska and Ivry, 2016) and it is also known to play a notable role in predictive timing, i.e., the ability to develop temporal expectations about upcoming events based on previous temporal information (Bastian, 2006; Schwartze and Kotz, 2013, 2016; Kotz et al., 2014). Predictive timing abilities are particularly useful when coordinating one’s action with rhythmic events such as music, which has a very regular and predictable structure (Dalla Bella et al., 2013). The cerebellum is therefore active in sensorimotor synchronization tasks (synchronized finger tapping) in adults (Rao et al., 1997; Jäncke et al., 2000; Stoodley et al., 2012) and in children (Rivkin et al., 2003; De Guio et al., 2012), but could also be very important when we are singing previously listened tunes, in particular when they are familiar, since it involves anticipating the rhythmic structure of the sequence to be sung.

Further support for the involvement of the cerebellum in music processing comes from studies of patients with cerebellar lesions, most of which have been conducted on patients with acquired lesions. First, sound duration perception deficits were found in adults with various cerebellar damages such as stroke, tumor or cerebellar degeneration (Ivry and Keele, 1989) and in children with cerebellar degeneration in the context of ataxia telangiectasia (Mostofsky et al., 2000). Such deficits have also been reported in temporal duration reproduction tasks in children after cerebellar tumor resection (Droit-Volet et al., 2013). Moreover, in addition to duration perception or reproduction deficits, rhythm perception and production has also been shown to be impaired in patients with cerebellar damages. According to an electroencephalography study, cerebellar lesions may have a specific impact on rhythm processing when played at a fast pace as it require more resources for accurate encoding of events (Nozaradan et al., 2017). This complements studies that have shown deficits in tempo change detection tasks in adult patients with cerebellar damage (Molinari et al., 2003; Schwartze et al., 2016). Studies involving rhythmic production have also shown that adult and children patients with cerebellar lesions exhibit greater variability in tapping tasks than control participants, both in spontaneous tapping and in tapping synchronized with auditory sequences of isochronous stimuli or in adaptive tasks, consistent with a cerebellar role in predictive timing (Ivry and Keele, 1989; Provasi et al., 2014b; Schwartze et al., 2016). However, some studies reported also preserved rhythm perception abilities in acquired cerebellar disorder (Grube et al., 2010a; Provasi et al., 2014b). Indeed, children with acquired cerebellar lesions following tumor resection showed preserved abilities in a tempo discrimination task (Provasi et al., 2014b). Similarly, adults with spino-cerebellar ataxia performed similarly to controls on rhythmic tasks despite deficits for absolute timing perception of single intervals (Grube et al., 2010a). The question of whether the temporal deficits related to a cerebellum lesion only concern durations or extend to rhythmic skills is therefore still controversial.

To our knowledge, only one clinical study has investigated both rhythmic and melodic musical perception in a group of adult patients with cerebellar acquired disorders (Tölgyesi and Evers, 2014) in the context of a degenerative affection (i.e., Machado-Joseph disease) or of cerebellar stroke. All the patients were significantly impaired in melody perception tested with a melody comparison task, and the group of patients with cerebellar degeneration also showed impairment in the ability to recognize familiar melodies. In addition, the authors proposed a rhythm reproduction task in which participants were asked to reproduce short rhythmic sequences by tapping with a pen on a table while the examiner scored the correctness of the rhythm and the meter. Patients with cerebellar damage performed similarly to controls in reproducing the rhythmic pattern but had significantly lower scores for the meter component, suggesting that rhythm production deficits were due to difficulties in maintaining a stable tempo rather than in reproduction rhythmic pattern *per se*. Melodic production was not investigated in this study, so it is not known whether this ability is affected in these patients. Singing, a widespread popular practice, most often requires the production of both rhythmic and melodic patterns (Dalla Bella et al., 2007). Only one pilot study has recently addressed the issue of singing abilities in adult patients with cerebellar disorders (Zúñiga et al., 2020). In this case study, the authors examined the speech and singing abilities of two ataxic patients with cerebellar dysarthria, a speech disorder resulting from motor coordination impairments in the context of cerebellar damage (Ackermann et al., 1992). For both patients, the authors reported alterations in basic motor processes concerning breathing, phonation and prosody. Moreover, speech and singing abilities were related to the degree of dysarthria, highlighting the potential influence of cerebellar coordination disorders on music production abilities. The patients’ music perception abilities were not assessed in this study.

The aforementioned clinical studies were conducted in patients with acquired cerebellar lesions and, to our knowledge, very few studies to date have explored some partial aspects of musical abilities in developmental cerebellar anomalies, which are characterized by cerebellar disorders present from birth or very early in development. None of them has explored in a comprehensive way the musical abilities by looking at both the melodic and rhythmic sides as well as the perceptual and productive aspects. In a study of children with DCA associated with spina bifida meningomyelocele, Dennis et al. (2004) reported impaired duration but preserved pitch perception around 3,000 Hz using discrimination tasks. The authors also reported greater variability in a synchronization-continuation tapping task when children were required to continue tapping rhythmically after the rhythmic stimulus had stopped, suggesting that predictive timing could be affected in these patients. Rhythm perception has also been studied in this same population (Hopyan et al., 2009). The authors showed that in comparison to healthy controls, children with DCA displayed a deficit in rhythm perception when comparing pairs of non-syncopated rhythms produced by drum sound. Dennis et al. (2009) replicated these findings in a larger sample and showed an association between rhythm perception deficits and cerebellar volumes. More recently, in a multiple-case study designed to evaluate the therapeutic benefit of dance interventions, deficits in sensorimotor synchronization to the metronome and/or music were found in six out of seven children with DCA (Bégel et al., 2022a). Taken together, these studies suggest that DCA patients present a musical deficit of rhythm that could be at least partially linked to predictive timing difficulties. However, none of these studies explored melody in addition to rhythm perception in the same children with DCA, and the only production component explored was synchronized finger tapping.

The primary objective of this novel study was therefore to examine music perception and production (singing) abilities in children with DCA. A secondary objective was to explore, in both groups of participants, the relationship between singing abilities and rhythmic and melodic perceptual abilities, as well as the relationship between singing and oro-bucco-facial praxis which is typically impaired in cerebellar syndrome. For this purpose, sixteen children with DCA were compared to 37 healthy controls matched for age and non-verbal intelligence. Music perception abilities were assessed using the abbreviated version of the Montreal Music Ability Assessment Battery (MBEMA) (Peretz et al., 2013). This battery assesses melodic and rhythmic perception, as well as musical memory. Music production abilities were evaluated using two singing tasks, a pitch matching task and a melodic reproduction task (Clément et al., 2015). The melodic reproduction task involved either familiar or unfamiliar stimuli. In addition, oro-bucco-facial praxis was assessed using the Hénin-Dulac test in which children had to reproduce different movements involving the oral region (Hénin, 1981).

We expected that children with DCA would perform worse than healthy controls in both perception and music production. We also expected that patients’ deficits would be more pronounced in rhythmic perception than in melodic perception. For the melodic reproduction task, as observed by Clément et al. (2015), we expected a condition effect in the control group, with an advantage for singing melodies from familiar songs over melodies from familiar tunes, and an advantage for singing the latter over unfamiliar melodies. Finally, we hypothesized positive correlations between perceptual abilities (melodic or rhythmic) and melodic singing on the one hand, and between oro-bucco-facial praxis abilities and melodic singing on the other hand. Because individuals with DCA have different types of cerebellar anomalies and this is a source of principled variability within the group, we were also interested in performing individual analyses to highlight potential differences in profiles or potential dissociations.

## Materials and methods

### Participants

#### Patients

Twenty-nine patients with developmental cerebellar anomalies (DCA group) were first selected at the Centre national de référence Malformations et Maladies Congénitales du Cervelet (C2M2C, Lille University Hospital). In order to isolate the specific contribution of the cerebellum to musical abilities, the following exclusion criteria were applied: (1) supratentorial abnormalities visible on MRI, (2) progressive cerebellar pathology, (3) epileptic seizures or febrile convulsions, (4) uncorrected auditory deficit, (5) intellectual disability determined by an intelligence quotient (IQ) < 70, measured by the Wechsler Intelligence Scale (Wechsler, 2005, 2016). After applying the exclusion criteria, sixteen children with congenital cerebellar ataxia participated in this study (nine boys and seven girls, aged from 8.0 to 13.0 years). All patients had a clinical cerebellar syndrome evaluated during a neurological examination performed by a specialist neuropediatrician and they all benefited from a neuropsychological evaluation. Patient 1 was diagnosed with cerebellar ataxia in the context of a Coffin-Siris syndrome linked to a mutation in the ARID1B gene, Patient 11 in the context of a Joubert syndrome linked to mutations in the CC2D2A gene, and Patient 15 in the context of mutations in the CACNA1A gene. For the thirteen other patients, the diagnosis of DCA was of unknown etiology. In 12 patients, cerebellar ataxia was associated with abnormalities observable with magnetic resonance imaging (MRI 3 Tesla), while in the other four patients, no abnormality was visible on MRI. At the moment of the study, 10 of the patients were or had been followed up by a speech therapist for speech and/or language difficulties (Patient 1, 3, 4, 10, 11, 12, 13, 14, 15, 16). A detailed description of patient characteristics is available in Supplementary Table 1.

#### Controls

A group of 37 typically developing children (CONTROL group; 13 boys and 24 girls, aged 7.8–13.1 years) was recruited from different schools in Hauts-de-France. None of the children in the control group had any known neurological, psychiatric or developmental disorder or hearing impairment at the time of the study. In order to check their general functioning, the children in the control group were assessed with four subtests of the WISC IV battery (Wechsler, 2005). These subtests assessed non-verbal intelligence (Matrix), working memory (Digit Span), and processing speed (Coding and Symbol Search). No children with deficits in any of these tests were included in the study.

The two groups of children were matched for age and non-verbal intelligence (standard score on the Matrix subtest). The CONTROL and DCA groups did not differ in either age [*t*_(51)_ = −0.402; *p* > 0.05] or non-verbal intelligence score [*t*_(51)_ = −0.856; *p* > 0.05]. In both groups, the first language was French for all children. None of the children had received more than 1 year of formal music or dance training. All participants and their parents signed informed consent in accordance with the Declaration of Helsinki to participate in the study.

### Material and procedure

#### Musical perception assessment

Musical perception abilities were assessed using the short version of the MBEMA (Peretz et al., 2013). It consists of three subtests, each with twenty items. In the first two subtests, each item consists of a target melody and a comparison melody separated by an interval of 1.5 s, and the child must decide whether they are similar or not. The comparison melodies can differ either melodically (subtest ‘‘Melody’’) or rhythmically (subtest ‘‘Rhythm’’). In the last subtest ‘‘Memory’’ the child hears 20 melodies, 10 of which were part of the two previous subtests and has to decide whether the melody has been heard before or not. The stimuli used in the abbreviated MBEMA can be downloaded from the website of Isabelle Peretz.^1^

#### Musical production assessment

Each participant played a computer-game “goose game,” created by Clément et al. (2015), against the experimenter. Through this game, the children and the experimenter were prompted to perform two singing tasks: a pitch-matching task and a melodic reproduction task. The game was designed so that the children would always win, and so that each child would have the same number of productions. The order of all the stimuli to be sung during the game (single notes and melodies) was randomized and identical for all the children. Presenting these singing tasks as a game allowed for motivating competition between the child and the experimenter.

In the “pitch-matching” trials, a piano note was played twice, and after each presentation, the participant had to sing back the note on the syllable/*la/*. Over the entire game, children had to reproduce all 12 degrees of the tempered scale from C4 (f0 = 261.23 Hz) to B4 (f0 = 493.88 Hz). Stimuli were produced using the virtual instrument “Steinway Grand Piano” in Apple Logic Pro 9 software and had an average duration of 1.4 s.

In the “melodic reproduction” trials, a short melody was played twice on the piano, and the participants had to sing it on the syllable/*la/* after each presentation. There was a total of six melodies to sing: two familiar songs melodies that are usually associated with lyrics and learnt in early childhood (FAM-SONG condition: “Brother John,” “Au Clair de la Lune”), two familiar melodies from movies or cartoons, not associated with lyrics and not learned explicitly in childhood (FAM-TUNE condition: “Pink Panther theme,” “Mission: Impossible”), and two unknown melodies composed for the study (UNFAM-TUNE condition: “Unknown A,” “Unknown B”). The familiar melodies consisted of the first musical phrase of the main theme, 4–5 bars in length. The unknown melodies were similar in length, had the same rhythmic and melodic complexity, and used the same pitch range as the familiar melodies. The scores of the melodies are available as Supplementary Figure 1. In the FAM-SONG and FAM-TUNE (familiar melodies) conditions, the title of the melody was announced to the child before the listening to the sample. After the experiment, we verified that all children indeed knew the familiar melodies by asking them about their knowledge of where the melodies came from and whether they had heard them often before. Stimuli were produced with the same configuration as for the single notes. All melodies were created with a fixed MIDI velocity of 80 and a tempo of 100 bpm, resulting in an average duration of 7.02 s (*SD* = 1.9 s).

During the game, all the stimuli were presented through headphones (Sennheiser HD 265 linear), and all the sung productions were recorded with a Zoom H2 digital audio recorder (uncompressed WAV file type, 16-bit/44.1 kHz) placed in front of the children.

#### Oro-bucco-facial praxis assessment

Each participant’s oro-bucco-facial praxis was assessed with the clinical test of oro-bucco-facial praxis (Hénin, 1981). In this test, the experimenter describes and performs a series of movements of the lips, tongue, cheeks, and mandibles, or eyes and forehead, and participants are asked to reproduce each of them successively. Each movement correctly reproduced in one or two trials was considered a success.

All children were assessed individually in a quiet room. Music perception and production abilities were tested, followed by an assessment of oro-bucco-facial praxis. The protocol lasted approximately 45 min. The children were offered a break between each task.

### Measures

#### Musical perception

For the three tests of the abbreviated MBEMA (“Melody,” “Rhythm,” and “Memory”), a score out of 20 is assigned to the child according to the number of correct answers provided (Peretz et al., 2013).

#### Musical production

All sung productions (single notes and melodies) were extracted from the continuous recording using Audacity version 2.2.1 recording and editing software (Audacity Team, 2019) for evaluation. To measure the pitch-matching accuracy, the pitch of each note was calculated using the algorithm provided by the “Pitchtrack” function of the phonTools package (Barreda, 2015) for R software (R Core Team, 2021). For each note, “Pitchtrack” measures the fundamental frequency every 10 ms. Of these measurements, only those of quantiles 2 and 3 are kept and averaged. The pitch obtained in Hertz (Hz) is compared to the theoretical pitch of the expected notes. This comparison allows for octave transpositions: if a participant had to sing an “A” at 440 Hz and sings a note at 830 Hz, the lower octave will be used (830/2 = 415 Hz) and the error is measured between 415 Hz and the target of 440 Hz. This error is expressed in cents (hundredths of semitones).

To assess melodic reproductions, as in Clément et al. (2015), we chose a subjective assessment method rather than a computerized analysis because some singing productions of children with DCA were severely impaired, so much so that it was not possible to recognize the original melody. Subjective rating is a method that has been validated and has been found to have a high congruence with objective measurements of vocal accuracy (Larrouy-Maestri et al., 2013). All melodic reproductions of the children were sorted by tune and presented in a random order to 10 healthy judges (five males and five females; age: mean = 25.9 years, *SD* = 2.2 years; musical background: mean = 2.4 years, *SD* = 3.0 yrs) using PsychoPy software (Peirce et al., 2019). For each trial, they heard the piano example, followed by a child’s song. They were then asked to give an overall score to the child’s production by clicking on a continuous scale from 0 (very poor performance) to 10 (very good performance), considering all musical parameters such as pitch and rhythm. They were also explicitly asked to ignore any global transposition of the melodies. Each judge was asked to rate all 636 productions (53 children; two trials per tune, six tunes). To avoid fatigue effects, the ratings were conducted in several sessions spread over several days. The judges were unaware of the presence of recordings of children with cerebellar disorders and were therefore blind to group affiliation. The ratings of the 10 judges were then averaged for each child’s production.

#### Oro-bucco-facial praxis

For each movement successfully completed in one or two trials, the participant receives one point. For each of the four movement categories assessed (“lips”, “tongue”, “cheeks and mandibles”, “eyes and forehead”), the score is transformed to a score out of 10 that can be compared to norms. We then calculated an overall Oro-bucco-facial praxis score for each child by adding these four scores, obtaining an overall score out of 40.

### Statistical analyses

Data processing and statistical analyses were performed using R studio software version 4.1.2 (R Core Team, 2021). To investigate the relationship between music perception abilities (MBEMA scores) and group (DCA, CONTROL), and the relationship between melody singing ability and group, linear mixed models were fit using the packages Lme4 version1.1-29 (Bates et al., 2015) and LmerTest version 3.1-3 (Kuznetsova et al., 2017). For all models, a Satterthwaite adjustment was used to compute the degrees of freedom. *Post-hoc* tests were computed with Holm–Bonferroni correction using the emmeans package version 1.7.4-1 (Lenth et al., 2022). Additionally, inter-rater agreement was assessed for the subjective ratings of the melodies, using Intraclass Correlation Coefficient (ICC) computed with the SimplyAgree R package version 0.0.3 (Caldwell, 2022).

Spearman correlation tests were used to explore the relationships between music perception, singing, and oro-buccofacial praxis, assuming that these relations may not be linear. To avoid an increase in Type I error due to multiple testing, Holm-Bonferroni corrections were applied to the results.

Individual analyses were performed in order to estimate the proportion of children with DCA who were impaired in each task and to examine, for each patient, the presence of a concomitant deficit in the Melody and Rhythm subtests of the MBEMA and/or the Oro-Bucco-Facial praxis. As the sample of the CONTROL group (*n* = 37) did not allow us to use the z-score method, we used the Bayesian Test of Deficit proposed by Crawford et al. (2011) allowing us to compare each patient to the CONTROL group, controlling for the effects of age as a covariate. These comparisons were done using the “BTD_cov” software published by the authors.

## Results

### Musical perception

**FIGURE 1:**
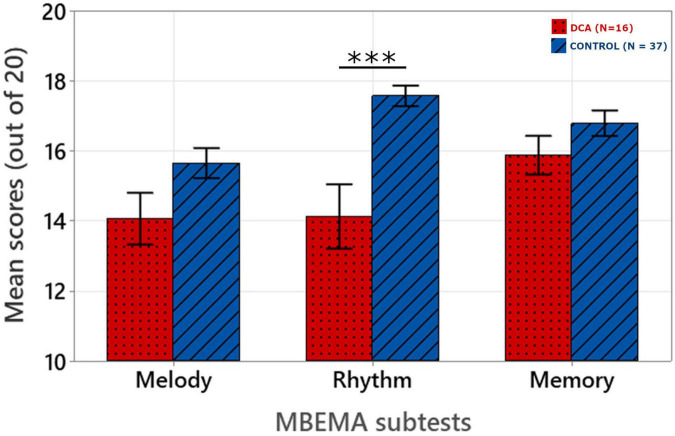
Mean scores obtained for each subtest of the MBEMA. Origin is set at chance level (10/20). The error bars correspond to the standard error of the mean. ***p < 0.001.

The mean scores on the three MBEMA subtests are presented in Figure 1. A linear mixed model was built to analyze MBEMA scores as a function of participant group and subtest. A “full model” was built including Group (DCA, Control), Subtest (Melody, Rhythm, Memory) and the Group-by-Subtest interaction as fixed effects together with a random intercept for Subjects.

This full model was compared to four reduced models by successively removing interaction term and then either Group or Subtest predictors. All four models were first fitted with maximum likelihood method (ML) to enable comparisons. Based on Likelihood Ratio Tests (LRT) and Akaike’s Information Criterion (AIC) (Akaike, 1974; Bozdogan, 1987), the full model was identified as the best fitting model. Details of the model comparisons are available in Supplementary Table 2.

The final model was then fit with Restricted Maximum Likelihood method (REML) (Marginal *R*^2^ = 0.194, Conditional *R*^2^ = 0.574). Visual inspection of the residuals plots as well as the random effects deviation plots did not reveal any obvious deviation from normality. Equation and coefficient of the model are presented in Table 1.

**TABLE 1:** Summary of the final linear mixed model for MBEMA analyses.

Final model equation: MBEMA_score ∼ 1 + group + subtest + group:subtest + (1 | Subject)						

Fixed effects						

	Estimate	Std. Error	95% CI lower	95% CI upper	t	*p*
Intercept	16.67	0.33	6.024	17.309	5.84	<0.001
GroupCEREB	−1.98	0.60	−3.149	−0.810	−3.38	0.002
Subtest 1	−1.02	0.24	−1.493	−0.543	−4.20	<0.001
Subtest 2	0.18	0.24	−0.357	0.591	0.48	0.630
GroupCEREB × Subtest 1	0.39	0.44	−0.471	1.257	0.89	0.376
GroupCEREB × Subtest 2	1.07	0.44	0.207	1.934	2.43	0.017

		**Random effects**				

		Variance			Std Deviation	
Participant (intercept)		2.89			1.70	

		**Model fit**				

		Marginal			Conditional	
*R* ^2^		0.194			0.573	

Sum-coding contrast method was used. p-values for fixed effects have been calculated using Satterthwaites approximations. Confidence Intervals have been calculated using the Wald method.

Fixed effects omnibus Anova (Type III) was performed with Satterthwaite method for degrees of freedom. This revealed an significant Group by Subtest interaction [*F*(2, 102) = 5.91; *p* = 0.004] as well as significant main effects of Group [*F*(1, 51) = 11.00; *p* = 0.002] and Subtest [*F*(2, 102) = 7.75; *p* < 0.001].

*Post hoc* analysis with Holm-Bonferroni correction revealed that patients with DCA obtained significantly lower scores than healthy controls on the Rhythm subtest (*p* < 0.001) whereas there was no significant difference between the two groups for the Melody (*p* = 0.209) and Memory (*p* = 0.067) subtests.

### Musical Production

#### Pitch-matching

A mean pitch error was computed for each group by averaging the absolute pitch errors. Since the assumption of normality of the distributions was not validated, the Mann Whitney *U*-test was used to compare these means. There was no significant difference between the mean pitch errors of DCA and CONTROL participants (*W* = 228; *p* = 0.097; mean error: DCA group = 233 cents; CONTROL group = 198 cents).

#### Melodic singing

**FIGURE 2:**
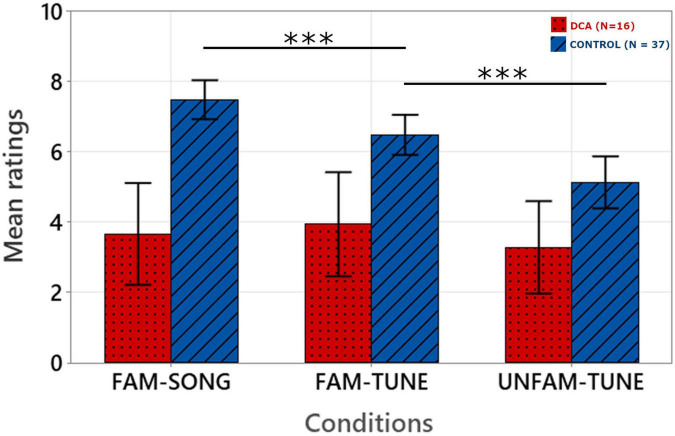
Mean ratings on the melodic reproduction task as a function of experimental conditions. The error bars correspond to the standard error of the mean. Ratings were given by 10 blind judges on a continuous scale from 0 (very poor performance) to 10 (very good performance) and were then averaged. ***p < 0.001.

Figure 2 shows the average ratings given by judges in the melodic reproduction (singing) task for the two groups of participants (DCA and CONTROL) as a function of condition (FAM-SONG; FAM-TUNE; UNFAM-TUNE). Ratings of the 10 blind judges were averaged for each children’s production.

We used a linear mixed model to evaluate the effect of group on the ability to sing melodies. There were two melodies per condition, and each of the 53 children sang twice for each melody, so there were 636 observations.

A random slope and intercept model was built with a by-subjects random intercept as well as a by-subject random slope for the effect of condition, assuming correlation between the random intercept and slope. Group (DCA, CONTROL), Condition (FAM-SONG, FAM-TUNE, UNFAM-TUNE) and the interaction between Group and Condition was entered as fixed effects. This full model was compared to the reduced model with no interaction, and to the two reduced models with only group or condition as a fixed effect. We also compared the full model to a random intercept by subject model with the same fixed effects. For comparisons, models with different fixed effects were fit with ML method and model with different random effects were fit with REML method. Model comparisons base on LRT and AIC identified the full model with random slope and intercept as the best fitting model. Details of the model comparisons are available in Supplementary Table 3.

The final model was then fit with REML (Marginal *R*^2^ = 0.279 Conditional *R*^2^ = 0.794). Visual inspection of the residuals plots as well as the random effects deviation plots did not reveal any obvious deviation from normality. Equation and coefficients of the model are presented in Table 2.

**TABLE 2:** Summary of the final Linear Mixed Model for the melodic reproduction task analyses.

Final model equation: melody_rating ∼ 1 + group + condition + group:condition + (1 + condition | Subject)						

Fixed effects						

	Estimate	Std. Error	95% CI lower	95% CI upper	t	*p*
Intercept	6.35	0.31	5.745	6.962	2.47	<0.001
GroupCEREB	−2.73	0.56	−3.838	−1.624	−4.84	<0.001
Condition 1	1.11	0.16	0.810	1.418	7.17	<0.001
Condition 2	0.12	0.13	−0.132	0.369	0.92	0.360
GroupCEREB × condition 1	−1.08	0.28	−1.635	−0.527	−3.82	<0.001
GroupCEREB × condition 2	0.19	0.23	−0.262	0.650	0.83	0.409

		**Random effects**			

		Variance		Std Deviation	
Participant (intercept)		3.43		1.85	
condition1		0.67		0.79	
Condition2		0.33		0.57	

		**Model fit**			

		Marginal		Conditional	
*R* ^2^		0.279		0.794	

Sum-coding contrast method was used. p-values for fixed effects have been calculated using Satterthwaites approximations. Confidence Intervals have been calculated using the Wald method.

Fixed effects omnibus anova (Type III) with Satterthwaite method for degrees of freedom highlighted an effect of Group [*F*(1, 51) = 23.381; *p* ≤ 0.001], Condition [*F*(2, 51) = 7.75; *p* < 0.001] and a Group by Condition interaction [*F*(2, 51) = 5.91; *p* = 0.001].

*Post hoc* analysis with Holm-Bonferroni correction showed that in the CONTROL group, performance was significantly better in the FAM-SONG condition than in the FAM-TUNE condition (*p* < 0.001), which were themselves significantly better than in the UNFAM-TUNE condition (*p* < 0.001). In contrast, in the DCA group, no significant difference was found between the FAM-SONG and FAM-TUNE condition (*p* = 0.753) and between the FAM-TUNE and UNFAM-TUNE condition (*p* = 0.441).

We also verified inter-rater reliability of the production ratings using ICC based on a single rater, absolute agreement, two-way random effects model (Koo and Li, 2016). We found a moderate to good agreement between the 10 judges [ICC(2, 1) = 0.7529, lower bound = 0.7238, upper bound = 0.7794].

### Correlations between musical perception and production

**FIGURE 3:**
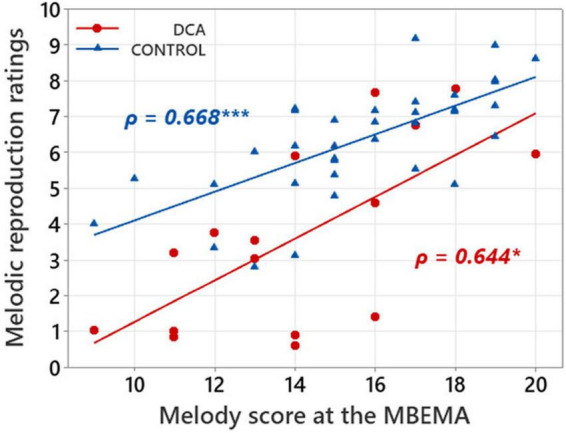
Scatterplot illustrating the correlations between mean ratings in melodic reproduction task and scores on the MBEMA Melody subtest. Regression lines are fitted for each group and the Spearman’s ρ are indicated. *p < 0.05 and ***p < 0.001.

**FIGURE 4:**
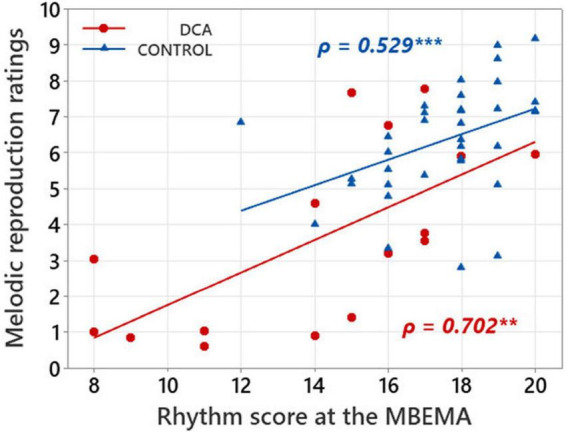
Scatterplot illustrating the correlations between mean ratings in melody singing and scores on the MBEMA Rhythm subtest. Regression lines are fitted for each group and the Spearman’s ρ are indicated. **p < 0.01 and ***p < 0.001.

Links between music perception and production abilities in each group of participants were explored with Spearman correlations tests between MBEMA Melody and Rhythm subtests scores and the average melodic singing scores. In each of the two groups of participants, we found two positive correlations between scores on the music perception subtests (Melody and Rhythm) of the MBEMA and performance in the melodic singing task (respectively, for the Melody subtest: DCA: ρ = 0.644, *p* = 0.011; CONTROL: ρ = 0.668; *p* < 0.001 and for the Rhythm subtest: DCA: ρ = 0.702, *p* = 0.002; CONTROL: ρ = 0.529; *p* < 0.001). Figures 3, 4 display the scatter plots of these correlations.

### Oro-bucco-facial praxis

We compared the global score of oro-bucco-facial praxis of the DCA and CONTROL groups. Mann Whitney *U*-test was used as the normality assumption was not validated. As expected, given their motor coordination disorders, the results showed that the DCA group scored significantly lower than the CONTROL group in the Oro-Bucco-Facial Praxia test (DCA: mean score = 31.7, *SD* = 7.92; CONTROL: mean score = 38.9, SD = 1.33; *W* = 58.0; *p* < 0.001).

We calculated correlations between the oro-bucco-facial praxis score and melodic singing performance, highlighting a strong positive correlation between the overall oro-bucco-facial praxis scores and mean scores on the melodic singing reproduction task in the DCA group (DCA: ρ = 0.728, *p* < 0.001) but not in the CONTROL group (ρ = 0.314, *p* = 0.087).

### Individual analyses

Bayesian Tests of Deficit (Crawford et al., 2011) revealed that eight out of sixteen patients with DCA, i.e., half of them, had a deficit in music perception, including seven patients with a rhythmic deficit and two patients with a melodic deficit (Table 3). Only one patient was impaired in both the Rhythm and Melody subtests of the MBEMA. Although all sixteen patients in the DCA group were able to sing the single notes correctly, 10 out of sixteen patients were impaired in the melodic singing task when compared to the children of the CONTROL group. These singing deficits were systematically associated with a deficit also found in music perception (rhythm and/or melody) and/or oro-bucco-facial praxis. Concerning the praxis, 12 out of sixteen patients had a very low score in the Oro-Bucco-Facial Praxis test. Finally, four out of the sixteen patients with DCA had no deficit in any of the tasks of our study.

**TABLE 3:** Summary of the individual analyses results for each task.

		*P*-Values at the Bayesian test of deficit					

	Musical production (Singing tasks, Clément et al., 2015)			Musical perception (MBEMA short version, Peretz et al., 2013)			Oro-Bucco-Facial Praxis (Hénin-Dulac Test, Hénin, 1981)
	
	Pitch-matching task		Melodic reproduction task	Melody subtest	Rhythm subtest	Memory subtest	
Patient 1	0.110		0.001	0.007	<0.001	0.472	<0.001
Patient 2	0.401		0.259	0.137	0.240	0.169	0.085
Patient 3	0.398		0.434	0.317	0.331	0.074	0.036
Patient 4	0.120		0.001	0.440	0.077	0.357	<0.001
Patient 5	0.393		0.087	0.423	0.011	0.138	<0.001
Patient 6	0.062		<0.001	0.245	0.021	0.495	<0.001
Patient 7	0.213		0.002	0.464	0.003	0.408	<0.001
Patient 8	0.407		0.286	0.317	0.210	0.225	0.202
Patient 9	0.397		0.169	0.394	0.101	0.343	0.372
Patient 10	0.333		0.002	0.109	<0.001	0.441	0.001
Patient 11	0.078		0.020	0.081	0.198	0.441	0.040
Patient 12	0.381		0.032	0.230	<0.001	0.374	<0.001
Patient 13	0.093		0.496	0.414	0.109	0.153	0.496
Patient 14	0.107		0.042	0.069	0.326	0.268	<0.001
Patient 15	0.082		0.001	0.054	<0.001	0.056	<0.001
Patient 16	0.081		0.020	0.033	0.159	<0.001	<0.001

Each patient was compared to the entire control group, controlling for age as a covariate for each test. The null hypothesis is that the case’s score is an observation of the control population’s scores. The p-value calculated is also the estimated proportion of controls with the same value on the covariate that are expected to obtain a lower score than the case (Crawford et al., 2011). All significant p-values are highlighted in grey (α = 0.05).

The aim of these individual analyses was to explore possible dissociations between perception and production. Interestingly, three patients (Patient 4, 11, 14) were impaired in the melodic singing task without having an associated deficit in music perception, but these three patients had deficits in oro-bucco-facial praxis. Conversely, one patient (Patient 5) had a perceptual deficit in the Rhythm subtest associated with a deficit in the Oro-Bucco-Facial praxis, but without any singing impairment, and another patient had a deficit in the Oro-Bucco-Facial praxis without any deficit in music perception or singing (patient 3). The two patients with a perceptual deficit in the Melody subtest had a pathological performance in singing as well (patients 1 and 16).

## Discussion

The purpose of this study was to explore music perception and production abilities in children with DCA. Music perception abilities were assessed using the MBEMA (Peretz et al., 2013), and music production abilities were assessed using two singing tasks presented via a playful and motivating computer game created by Clément et al. (2015). In addition, oro-bucco-facial praxis was assessed using the Hénin-Dulac test (Hénin, 1981). Results showed that children with DCA performed lower in rhythm perception and in singing short melodies, as well as in the oro-bucco-facial praxis test.

Regarding the music perception tasks, our mixed model analyses and *post hoc* tests showed that children with DCA scored significantly lower than healthy control children in the rhythm subtest of the MBEMA. In contrast, we did not find any significant difference between the two groups of participants in the melody comparison task or in the memory subtest of the MBEMA. These results bring further evidence of the important role of the cerebellum in rhythm processing already suggested by neuroimaging studies (Parsons, 2001; Chen et al., 2008; Thaut et al., 2014) and by studies showing rhythmic deficits in patients with cerebellar damages (Tölgyesi and Evers, 2014; Schwartze et al., 2016; Bégel et al., 2022a). In addition, these results provide a developmental perspective that has been little studied to date, suggesting a cerebellar role in the development of rhythmic abilities in children. Our findings complement those obtained in the two studies that reported rhythmic perception deficits in children with DCA in the specific case of spina bifida meningomyelocele (Dennis et al., 2009; Hopyan et al., 2009). Further, our results highlight the specificity of rhythmic perceptual deficits compared to melodic deficits and thus suggest that the musical difficulties experienced by children with DCA are not global. Our results also extend these findings of rhythmic deficits to other conditions with DCA, strengthening the evidence for a cerebellar role in the development of rhythm perception. The cerebellum is part of a distributed network that enables timing processes, including basal ganglia, the motor and premotor cortices (Grahn and Brett, 2007; Coull and Nobre, 2008; Coull et al., 2011; Breska and Ivry, 2016; Bareš et al., 2018). Its involvement is notable in predictive timing processes (Bastian, 2006; Schwartze and Kotz, 2013; Kotz et al., 2014; Schwartze et al., 2016) and in the neural tracking of beat and rhythm (Nozaradan et al., 2017). While the basal ganglia and associated cortico-striato-thalamo-cortical circuits seem to be specifically involved in rhythm processing that suppose high demand on internally generated beat, the cerebellum is thought to work in parallel to this circuit and contribute to this process via a precise encoding and rapid transmission of an event-based representation of the temporal structure (Schwartze and Kotz, 2013, 2016; Nozaradan et al., 2017). The rhythm subtest of the MBEMA involves pairs of short melodies that are likely to differ in the duration of two adjacent tones but maintain the same meter and tempo. Thus, this task requires rhythmic grouping skills that rely on a precise encoding of the temporal events, and require the ability to correctly predict the onset of upcoming event based on previous information. This task could therefore particularly involve the cerebellum. This task also requires working memory, and our findings are in line with studies that suggest a specific involvement of the cerebellum in working memory for rhythms and for time interval embedded in rhythmic sequences (Jerde et al., 2011; Teki and Griffiths, 2016).

Moreover, individual analyses revealed heterogeneity within the patient group. While the group of patients performed significantly lower in rhythm perception than the group of healthy control children, of the sixteen patients, only eight were impaired in at least one subtest of music perception, including seven patients with a deficient score in rhythm perception. Heterogeneity is commonly reported in neurodevelopmental disorders and especially in DCA (Tavano et al., 2007; Jissendi-Tchofo et al., 2011), but the fact that half of the patients did not show any deficit in music perception is of particular interest. One explanation for this disparity in results may lie in the diversity of cerebellar anomalies within the patient group and the fact that rhythm processing involves specific parts of the cerebellum. Especially, in children with DCA associated to spina bifida meningomyelocele, Dennis et al. (2009) showed that rhythm perception deficits were associated with specific volumetric variations in the cerebellum. We did not investigate cerebellar volumes in this study, and it is therefore possible that children without rhythmic deficits have different volumetric variations. It might also be possible that some children have developed compensatory mechanisms. Cerebral plasticity is a phenomenon that is a source of variability in the development of children with neurodevelopmental disorders and could thus also explain part of the observed heterogeneity. Functional outcomes depend on several interacting factors, such as differences in genes, socioeconomic status, and differences in support and care (Dennis et al., 2013, 2014). In particular, cerebellar volumes and sensorimotor skills may vary with music or dance training from an early age (Baer et al., 2015; Nigmatullina et al., 2015). Thus, each child’s individual development may be influenced by many of these factors. It should also be noted that studies of adult cerebellar patients with acquired lesions have also reported controversial results regarding the involvement of the cerebellum in rhythm perception and prediction abilities (Grube et al., 2010a; Breska and Ivry, 2016, 2018, 2020). Future studies should therefore further investigate the individual variability of rhythmic skills in cerebellar disorders to better understand its source.

With regard to music production abilities, mixed model analyses revealed that children with DCA were impaired in singing short melodies. Our melodic singing task had three conditions: melodies were either those of familiar songs, familiar tunes, or melodies that were new and therefore unknown. Whereas control children showed superior performance on familiar songs and familiar tunes over unknown melodies, children with DCA did not benefit from prior knowledge of melodies. Singing disorders were expected because of the known involvement of the cerebellum in different processes of the vocal sensorimotor loop (Berkowska and Dalla Bella, 2009). Especially, it generates motor-to-auditory predictions (Knolle et al., 2012) and contributes to the prediction of self-initiated sounds (Knolle et al., 2013). The cerebellum is also involved in motor control of vocal muscles (Grabski et al., 2012) and in the auditory feedback control of vocal production (Li et al., 2019; Peng et al., 2021). Therefore, cerebellar anomalies can impair singing production at different levels.

The fact that children with DCA were impaired in melodic singing but were able to sing single notes as well as controls suggests that the singing deficit is revealed by the melodic nature of the task. Singing melodies requires not only the matching of desired pitches, but also the production of accurate rhythms, and therefore requires both rhythmic and melodic perception skills. As expected, in both groups, strong correlations were found between the MBEMA melody and rhythm subtests and the melodic singing scores. Given that children in the DCA group were mostly impaired in rhythm perception, they may have encountered difficulties in singing the melodies with a correct rhythm. Indeed, individual analyses revealed that six of the seven patients impaired in the rhythm subtest were also impaired in singing melodies.

In addition, motor impairments associated with cerebellar dysfunction (Ackermann et al., 1992; Mariën et al., 2014) could also have altered production of melodies, as singing involves a precise coordination of the vocal muscles, as well as the precise coordination of the muscles of the respiratory and phonatory apparatus. Within the patient group, a strong correlation was found between the score at the oro-bucco-facial praxis test (Hénin, 1981) and the mean rating at melodic singing. Moreover, our individual analyses revealed that all the children who were impaired in melodic singing were also impaired in oro-bucco-facial praxis. Ataxic dysarthria is part of the cerebellar syndrome and is mainly manifested by articulatory deficits and slowed speech tempo (Ackermann et al., 1992). It is often also accompanied by respiratory and phonatory disorder which can cause irregular alterations of voice quality and loudness (Mariën et al., 2014). Therefore, it is not surprising that patients who displayed a deficit in oro-bucco-facial praxis were also impaired in the melodic singing task. These findings can be related to those of a recent study (Zúñiga et al., 2020) which suggest links between dysarthria and singing abilities in two patients suffering a cerebellar stroke.

Taken together, our findings suggest an important role of the cerebellum in the development of singing abilities, with impairments found in patients on both the perceptive and motor components of the vocal sensorimotor loop. To further investigate the results, we examined individual performance on the different tasks of the experiment, which allowed us to highlight performance dissociations. Three patients (patients 4, 11, 14) had melodic singing deficits in the presence of preserved perceptual abilities, suggesting that good perceptual musical abilities are not sufficient for the development of singing skills. This dissociation had already been documented in the literature (Berkowska and Dalla Bella, 2009). However, these three patients had a deficit of the oro-bucco-facial praxis, which could explain the singing deficit despite the preservation of musical perception. On the other hand, interestingly, a patient with an oro-bucco-facial praxis deficit (patient 3), and another patient (patient 5, also impaired in rhythm) showed correct performance in the singing task despite the presence of oro-bucco-facial praxis deficit, suggesting that the development of the ability to sing melodies correctly is still possible in the presence of such praxis deficits. Finally, four patients did not show any deficits in any tasks. All these findings suggest that singing abilities could depend both on the development of musical perception as well as of the oro-buccal praxis. Different developmental trajectories are possible in the presence of DCA, which is probably due to the presence of compensation mechanisms in some cases.

Understanding of the mechanisms underlying musical deficits in relation to DCA in the present study is limited by some shortcomings. First, the subjective assessment procedure for melodic singing does not allow to distinguish between rhythmic or melodic impairment. As in a previous study by Clément et al. (2015), we chose this method because some of the patients’ productions were impaired to the point that they contained only a few notes and were not recognizable. To unravel the impacts of rhythmic, melodic, and praxis deficits on music production abilities, future studies could use paradigms with conditions that specifically vary these parameters or consider new methods of singing analysis. Furthermore, it is not clear which specific mechanisms of rhythmic perception and/or production are affected. This study therefore calls for a more comprehensive assessment of rhythmic perception, production, and rhythm-based prediction abilities in the presence of DCA, varying conditions such as tempo, meter, or syncopation. Finally, it is worth reminding that all participants had received less than 1 year of formal music or dance instruction. However, non-formal music practice may vary among participants, and future studies should control for music and dance exposure. Similarly, we were not able to clearly control the different care paths of the patients. However, these differences in care may have had an impact on the development and implementation of compensatory mechanisms, which could partly explain the observed heterogeneity. Further studies should also investigate the links between speech and language and musical deficits in children with DCA. To explore these mechanisms and the underlying brain substrates, neuroimaging studies could provide interesting information, both from a structural and functional perspective.

## Conclusion and implications

Overall, our results suggest that DCA are associated with deficits in the musical sphere, especially in rhythmic perception and melodic singing. Further research will be needed to unravel the links between rhythmic, praxis, and singing deficits found in children with DCA. The impairment of rhythmic abilities is consistent with studies that have shown that rhythmic impairments are common in neurodevelopmental disorders (Lense et al., 2021), particularly in dyslexia (Bégel et al., 2022b) and ADHD (Puyjarinet et al., 2017), for which an involvement of the cerebellum has been highlighted (Stoodley, 2016; Sathyanesan et al., 2019). Rhythmic deficits are often associated with cognitive and social deficits, and therapeutic approaches based on rhythm and audio-motor synchronization such as dance are a promising tool for children with DCA as they seem to improve rhythmic abilities as well as cognitive functions (Bégel et al., 2022a). Our study provides new evidence regarding the involvement of the cerebellum in singing abilities and may pave the way for music-based remediation including singing training. Moreover, the heterogeneity of musical performance in this population must be considered before considering such interventions. Indeed, the different profiles observed suggest that various mechanisms may be affected in DCA, which should lead to a detailed assessment of musical abilities in these patients in order to orient them toward the intervention that will be most adapted to their needs.

## Data availability statement

The raw data supporting the conclusions of this article will be made available by the authors, without undue reservation.

## Ethics statement

Ethical review and approval was not required for the study on human participants in accordance with the local legislation and institutional requirements. Written informed consent to participate in this study was provided by the participants’ legal guardian/next of kin.

## Author contributions

AG, SC, and DD contributed to the analysis of the results. AG and DD contributed to the writing of the manuscript. All authors approved the submitted version, contributed to the design, and implementation of the research.

## Footnotes

1
http://www.peretzlab.ca/
