# Corrigendum: Musical abilities in children with developmental cerebellar anomalies

**DOI:** 10.3389/fnsys.2022.1079004

**Published:** 2022-11-09

**Authors:** Antoine Guinamard, Sylvain Clément, Sophie Goemaere, Alice Mary, Audrey Riquet, Delphine Dellacherie

**Affiliations:** ^1^Univ. Lille, ULR 4072 – PSITEC – Psychologie: Interactions, Temps, Émotions, Cognition, Lille, France; ^2^CHU Lille, Centre de Référence Malformations et Maladies Congénitales du Cervelet, Lille, France; ^3^CHU Lille, Centre Régional de Diagnostic des Troubles d'Apprentissage, Lille, France

**Keywords:** cerebellum, developmental cerebellar anomalies, ataxia, music perception, music production, singing, children, rhythm

In the published article, there was an error in affiliations 1, 2, and 3. Instead of “^1^ Univ. Lille, ULR 4072 – PSITEC – Psychologie: Interactions, Temps, Émotions, Cognition, Lille, France, ^2^ CHU Lille, Centre Régional de Diagnostic des Troubles d'Apprentissage, Lille, France, ^3^ CHU Lille, Centre de Référence Malformations et Maladies Congénitales du Cervelet, Lille, France”, it should be “^1^ Univ. Lille, ULR 4072 – PSITEC – Psychologie: Interactions, Temps, Émotions, Cognition, Lille, France, ^2^ CHU Lille, Centre de Référence Malformations et Maladies Congénitales du Cervelet, Lille, France, ^3^ CHU Lille, Centre Régional de Diagnostic des Troubles d'Apprentissage, Lille, France.”

The authors apologize for this error and state that this does not change the scientific conclusions of the article in any way. The original article has been updated.

## Publisher's note

All claims expressed in this article are solely those of the authors and do not necessarily represent those of their affiliated organizations, or those of the publisher, the editors and the reviewers. Any product that may be evaluated in this article, or claim that may be made by its manufacturer, is not guaranteed or endorsed by the publisher.

