# Supplementary material for: Musical abilities in children with developmental cerebellar anomalies
Source: Front Syst Neurosci. 2022 Aug 18;16:886427. doi: 10.3389/fnsys.2022.886427 (PMC9436271; doi:10.3389/fnsys.2022.886427)
Supplement: Supplementary file 1 [file Table_1.docx]

***Supplementary Material***

**Supplementary Table 1 – Detailed characteristics of the patient group**

| **Patient** | **Genre** | **Age** | **MRI** | **Etiology** | **IQ** | VCI | PRI | VSI | FRI | WMI | PSI |
| --- | --- | --- | --- | --- | --- | --- | --- | --- | --- | --- | --- |
| 1 | F | 10,6 | Minor hypoplasia of the lower vermis and dysmorphic appearance of the upper vermis and cerebellar hemispheres | ARID1B mutation  (Coffin-Siris’ Syndrome) | **82** | 89 | NT | 81 | 88 | 88 | 83 |
| 2 | F | 13,0 | Normal | Unknown | **93** | 99 | 114 | NT | NT | 82 | 78 |
| 3 | F | 9,8 | Hypoplasia of the inferior vermis | Unknown | **93** | 112 | 88 | NT | NT | 88 | 86 |
| 4 | M | 10,5 | Enlarged fourth ventricle, enlarged cerebellar sulci | Unknown | **83** | 103 | 88 | NT | NT | 76 | 73 |
| 5 | M | 12,1 | Abnormal conformation of the cerebellar hemispheres, aspects close to dysplasia | Unknown | **71** | 81 | 84 | NT | NT | 67* | 71 |
| 6 | M | 10,8 | Normal | Unknown | **124** | 126 | 124 | NT | NT | 112 | 103 |
| 7 | M | 8,0 | Enlargement of the upper vermian fissures | Unknown | **91** | 89 | NT | 94 | 103 | 94 | 72 |
| 8 | M | 12,5 | Minor vermian dysgenesis | Unknown | **88** | 101 | 96 | NT | NT | 76 | 83 |
| 9 | F | 9,9 | Enlargement of the upper vermian fissures | Unknown | **109** | 118 | NT | 102 | 103 | 112 | 100 |
| 10 | M | 8,2 | Normal | Unknown | **86** | 108 | 88 | NT | NT | 79 | 76 |
| 11 | F | 12,7 | Vermian dysplasia and hypoplasia, malformation of the cerebellar peduncles (Molar Tooth Sign) | CC2D2A mutation  (Joubert’s Syndrome) | **73** | 88 | 77 | NT | NT | 67* | 81 |
| 12 | M | 9,3 | Moderate hypoplasia of the inferior vermis and retro-cerebellar cyst | Unknown | **91** | 99 | 109 | NT | NT | 73 | 86 |
| 13 | F | 11,9 | Atrophy around the primary sulcus of the superior vermis | Unknown | **89** | 82 | 107 | NT | NT | 82 | 100 |
| 14 | M | 11,0 | Moderate atrophy of the superior vermis | Unknown | **97** | 98 | 104 | NT | NT | 94 | 93 |
| 15 | M | 9,9 | Vermian atrophy | CACNA1A mutation | **81** | 95 | NT | 89 | 85 | 76 | 86 |
| 16 | F | 11,0 | Normal | Unknown | **84** | 86 | 102 | NT | NT | 67* | 93 |
| *Note*: IQ and major index scores were obtained from the neuropsychological assessment closest to our study. They were calculated using the WISC-IV or the WISC-V (patients 1, 7, 9 and 15). MRI= Magnetic Resonance Imaging; IQ = Intelligence Quotient; VCI = Verbal Comprehension Index, PRI = Perceptual Reasoning Index; VSI = Visual Spatial Index; FRI = Fluid Reasoning Index; WMI =Working Memory Index; PSI = Processing Speed Index. Note that in the WISC IV, the main indexes are VCI, PRI, WMI and PSI. In the WISC V, the main indexes are VCI, VSI, FRI, WMI and PSI. NT = not tested (the index does not exist in the battery used); * = deficient scores (<70) | | | | | | | | | | | |
