# Supplementary material for: Musical abilities in children with developmental cerebellar anomalies
Source: Front Syst Neurosci. 2022 Aug 18;16:886427. doi: 10.3389/fnsys.2022.886427 (PMC9436271; doi:10.3389/fnsys.2022.886427)
Supplement: Supplementary file 2 [file Table_2.docx]

Supplementary Material

# Supplementary Table 2

**Supplementary Table 2 – Mixed model comparisons for MBEMA score analysis**

| reduced model 1 | MBEMA_score ~ 1 + group + (1 \| subject) | | | | | | |
| --- | --- | --- | --- | --- | --- | --- | --- |
| reduced model 2 | MBEMA_score ~ 1 + subtest + (1 \| subject) | | | | | | |
| reduced model 3 | MBEMA_score ~ 1 + group + subtest + (1 \| subject) | | | | | | |
| full model | MBEMA_score ~ 1 + group + subtest + group:subtest + (1 \| subject) | | | | | | |
|  | **AIC** | **BIC** | **logLik** | **deviance** | **Chisq** | **Df** | ***p*** |
| reduced model 1 | 738.14 | 750.42 | -365.07 | 730.14 |  |  |  |
| reduced model 2 | 733.53 | 748.87 | -361.76 | 723.53 | 6.6179 | 1 | .010 |
| reduced model 3 | 725.17 | 743.58 | -356.59 | 713.17 | 10.3553 | 1 | .001 |
| full model | 717.55 | 742.11 | -350.78 | 701.55 | 11.6170 | 2 | .003 |
| *Note*: AIC = Akaike’s information criterion; BIC = Bayesian Information Criterion; logLik = Log-Likelihood; Chisq = Chi square; Df = Degrees of freedom  Models were fit with Maximum Likelihood method. | | | | | | | |
