# Supplementary material for: Musical abilities in children with developmental cerebellar anomalies
Source: Front Syst Neurosci. 2022 Aug 18;16:886427. doi: 10.3389/fnsys.2022.886427 (PMC9436271; doi:10.3389/fnsys.2022.886427)
Supplement: Supplementary file 3 [file Table_3.docx]

Supplementary Material

# Supplementary Table 3

**Supplementary Table 3 – Mixed model comparisons for melody reproduction score analysis**

| **Models with different fixed effects** | | | | | | | |
| --- | --- | --- | --- | --- | --- | --- | --- |
| reduced model 1 | Melody_rating ~ 1 + group + (1 + condition \| subject) | | | | | | |
| reduced model 2 | Melody_rating ~ 1 + condition + (1 + condition \| subject) | | | | | | |
| reduced model 3 | Melody_rating ~ 1 + group + condition + (1 + condition \| subject) | | | | | | |
| full model | Melody_rating ~ 1 + group + condition + group:condition  + (1 + condition \| subject) | | | | | | |
|  | **AIC** | **BIC** | **logLik** | **deviance** | **Chisq** | **Df** | ***p*** |
| reduced model 1 | 2468.8 | 2508.9 | -1225.4 | 2450.8 |  |  |  |
| reduced model 2 | 2462.9 | 2507.4 | -1221.5 | 2442.9 | 7.8938 | 1 | .005 |
| reduced model 3 | 2446.2 | 2495.2 | -1212.1 | 2424.2 | 18.7055 | 1 | < .001 |
| full model | 2436.5 | 2494.4 | -1205.2 | 2410.5 | 13.7170 | 2 | 0.001 |
| **Models with different fixed effects** | | | | | | | |
| Random intercept | Melody_rating ~ 1 + group + condition + group:condition + (1 \| subject) | | | | | | |
| Random slope & intercept | Melody_rating ~ 1 + group + condition + group:condition + (1 + condition \| subject) | | | | | | |
|  | AIC | BIC | logLik | deviance | Chisq | Df | *p* |
| Random intercept | 2533.7 | 2569.3 | -1258.9 | 2517.7 |  |  |  |
| Random slope & intercept | 2443.4 | 2501.4 | -1208.7 | 2417.4 | 100.27 | 5 | < .001 |
| *Note*: AIC = Akaike’s information criterion; BIC = Bayesian Information Criterion; logLik = Log-Likelihood; Chisq = Chi square; Df = Degrees of freedom  Models with different fixed effect were fit with Maximum Likelihood method, and models with different random effects were fit wis Restricted Maximum Likelihood method. | | | | | | | |
