# Supplementary figures and images for: Musical abilities in children with developmental cerebellar anomalies
Source: Front Syst Neurosci. 2022 Aug 18;16:886427. doi: 10.3389/fnsys.2022.886427 (PMC9436271; doi:10.3389/fnsys.2022.886427)

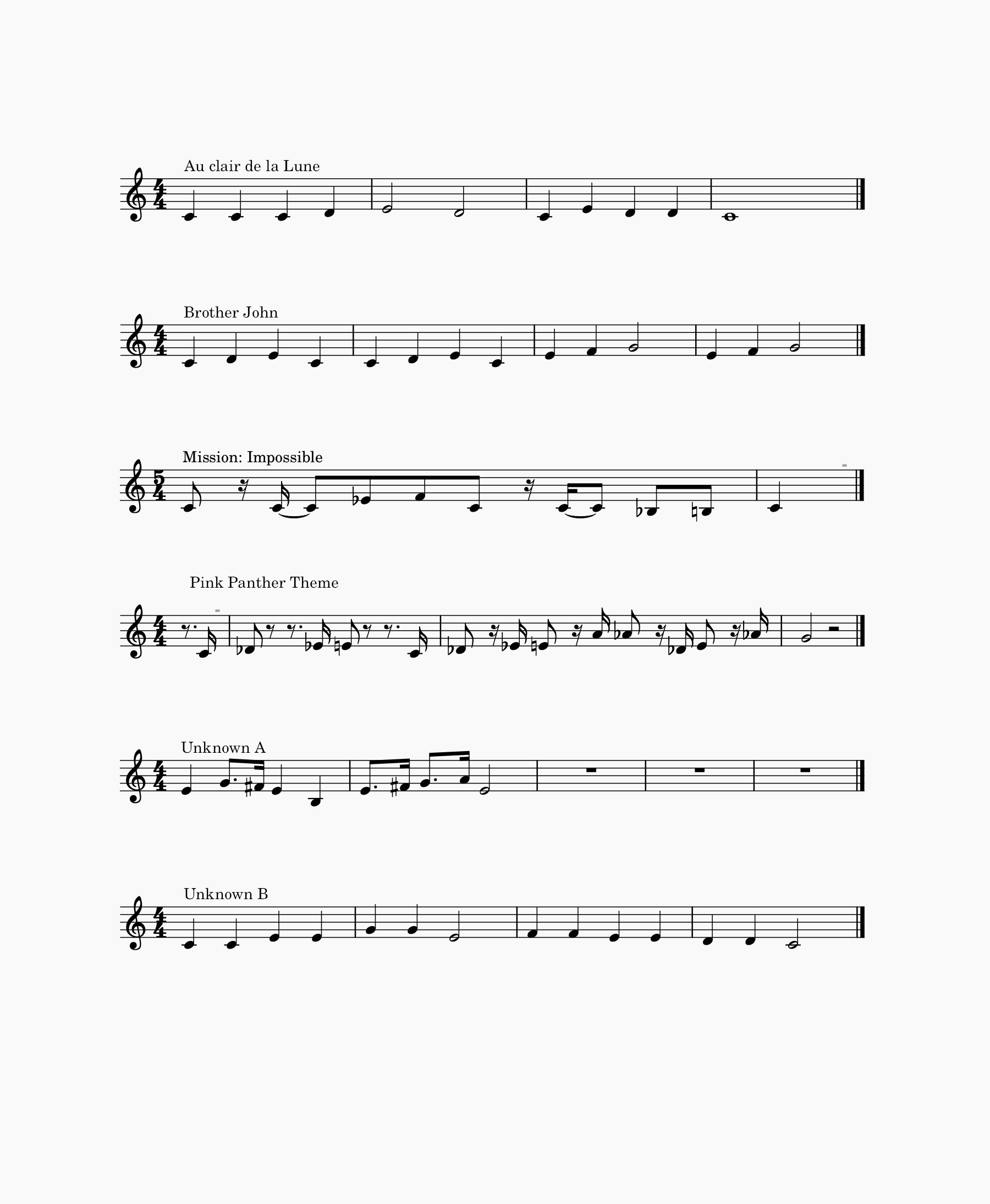

Supplement: Supplementary file 4 [file Image_1.jpeg]
